# Moderne Schilddrüsenchirurgie – das endokrin-chirurgische Verständnis des Operateurs und seine Verantwortung für Resektionsausmaß und Komplikationsrate

**DOI:** 10.1007/s10354-020-00750-5

**Published:** 2020-04-27

**Authors:** Michael Hermann, Elisabeth Gschwandtner, Max Schneider, Laura Handgriff, Rupert Prommegger

**Affiliations:** 1 Chirurgische Abteilung, Krankenanstalt Rudolfstiftung, Juchgasse 25, 1030 Wien, Österreich; 2 Klinische Abteilung für Thoraxchirurgie, Medizinische Universität Wien, Währinger Gürtel 18–20, 1090 Wien, Österreich; 3 Chirurgie, Sanatorium Kettenbrücke der Barmherzigen Schwestern, Sennstraße 1, 6020 Innsbruck, Österreich

**Keywords:** Schilddrüsenchirurgie, Thyreoidektomie, Schilddrüsenkarzinom, Recurrensparese, Hypoparathyreoidismus, Thyroid surgery, Thyroidectomy, Thyroid carcinoma, Recurrent nerve injury, Hypoparathyroidism

## Abstract

Die hohe Qualität der Schilddrüsenchirurgie impliziert ein endokrin-chirurgisches Verständnis des Operateurs mit dem Ziel einer bestmöglichen Ergebnisqualität. Das beinhaltet ein befundadäquates Resektionsausmaß und eine möglichst niedrige Komplikationsrate. Der Chirurg sollte frühzeitig in die Operationsindikation eingebunden sein und auch selbst die Diagnostik, speziell den Schilddrüsen- und Halslymphknotenultraschall, sowie die Interpretation der Schnittbild- und nuklearmedizinischen Verfahren beherrschen. Im Besonderen sollte er über zeitgemäße Radikalitätsprinzipien in der Chirurgie Bescheid wissen.

Bei der **gutartigen Struma** ist eine individualisierte Operationsstrategie anzuwenden: Solitärknoten können auch einer gewebeschonenden Knotenresektion unterzogen werden. Bei multinodulärer Knotenstruma ist nicht zwingend eine totale Thyreoidektomie notwendig, die Vermeidung eines permanenten Hypoparathyreoidismus hat Priorität. Bei Rezidivstrumen ist oft die einseitige Operation des dominanten Befundes zu bevorzugen. Auch besteht zunehmend der Trend, die Indikation zur Entfernung der Schilddrüsenlappen seitengetrennt zu stellen. Die Basedow Struma erfordert eine Thyreoidektomie. Auch die hypertrophe Thyreoiditis Hashimoto kann eine Operationsindikation darstellen.

Die Radikalitätsprinzipien bei **maligner Struma** haben sich ebenfalls deutlich gewandelt als auch die strenge Indikation zur Radiojodtherapie. Das gilt speziell für papilläre Mikrokarzinome und minimal invasive follikuläre Tumortypen. Selbst bei medullären Schilddrüsenkarzinom stehen die Radikalitätsprinzipien im Hinblick auf synchrone oder metachrone laterale Halsdissektion in Diskussion.

Der **Hypoparathyreoidismus** stellt derzeit das Hauptproblem in der radikalen Schilddrüsenchirurgie dar. **Recurrensparese** und **Nachblutung** sind durch die subtile Operationstechnik selten geworden. Spezielle extrazervikale Operationszugänge sind nach wie vor in der Erprobungsphase und unter strengen Studienbestimmungen nur Zentren vorbehalten. Die Radiofrequenzablation stellt für gewisse Läsionen wie Zysten und autonome Adenome bei chirurgischer Kontraindikation ein alternatives Ablationsverfahren dar.

## Summary

High quality thyroid surgery implies a surgeon with an endocrine-surgical understanding aiming at best possible outcome. This includes an appropriate extent of the resection and a low rate of complications. It is important that the surgeon is involved at an early stage being part of the decision process for or against partial or total thyroidectomy. Furthermore, the surgeon should not only be able to perform thyroid and cervical lymph node sonography, but also to be capable to interpret cross-sectional imaging modalities and nuclear medicine imaging procedures. A thorough knowledge of modern principles of radicality is essential.

Benign goiters require individualized surgical strategy: solitary nodules can be treated with a tissue-preserving selective nodular resection. However, a multinodular goiter does not necessarily require total thyroidectomy—prevention of a permanent hypoparathyroidism is of paramount importance. For recurrent goiters, removal of the dominant side and therefore, unilateral procedure is favored. Nowadays, there is an increasing tendency to set the indication for thyroid surgery separately for each lobe. Graves’ disease requires thyroidectomy, and occasionally, hypertrophic Hashimoto’s thyroiditis may also result in surgery.

The principles of radical surgical treatment of malignant goiters have changed significantly over the past few years and, so far, strict indication for postoperative radioiodine treatment is being reconsidered. This is especially relevant for papillary thyroid microcarcinomas and minimally invasive follicular tumors. Even the radical surgical treatment of medullary thyroid carcinoma, especially considering synchronous or metachronous lateral neck dissection, is currently under review.

Hypoparathyroidism is the most relevant complication in radical thyroid surgery and has devastating influence on the patients’ life quality. Nowadays, permanent recurrent laryngeal nerve injury and postoperative hemorrhage rarely occur due to subtle surgical techniques. Extracervical surgical access to the thyroid is still a matter of clinical trials and should be restricted to centers. Radiofrequency ablation is an alternative method for benign lesions or hyperfunctioning nodules in patients with high surgical risk.

## Einleitung

In der modernen Schilddrüsenchirurgie ist der Chirurg bereits in die OP-Indikation eingebunden, sofern es sich nach erster Diagnostik durch niedergelassene Ärzte, Nuklearmediziner, Endokrinologen oder Internisten nicht eindeutig um ein konservativ zu behandelndes Krankheitsbild handelt.

Die gemeinsame interdisziplinäre Einschätzung ermöglicht es dem Chirurgen, frühzeitig seine Stellungnahme einzubringen und im Bedarfsfall eine Operationsplanung und ein befundadaptiertes Resektionsausmaß vorzuschlagen. Dies bedeutet sowohl mit dem Patienten als auch mit dem behandelnden Diagnostiker die operativen Möglichkeiten bei dem jeweils betroffenen Krankheitsbild in Zusammenschau mit einer Risikoevaluierung bezüglich Morbidität des Eingriffs darzustellen. So steht beispielsweise außer Frage, dass eine fortgeschrittene Basedow-Erkrankung einer (möglichst) totalen Thyreoidektomie unterzogen werden muss. Weniger bekannt ist vielen zuweisenden Nuklearmedizinern und Endokrinologen, dass solitäre Knoten in der Schilddrüse bei entsprechend günstiger Lokalisation (und definitiv gutartiger Histologie) auch einer sparsamen funktionskritischen und gewebeerhaltenden Knotenresektion unterzogen werden können, ohne dass dabei ein erhöhtes Risiko für einen potenziellen späteren Re-Eingriff gegeben ist. Aktuelle Leitlinien geben einen Entscheidungskorridor vor, gemäß der betreffenden Leitlinie der operativen Therapie benigner Schilddrüsenerkrankungen kann mit dem Patienten in solchen Fällen ein individuelles Therapieziel vereinbart werden [1]. Über die Vor- und Nachteile einer gewebeerhaltenden parenchymsparenden Operation (Notwendigkeit der Komplettierungsoperation bei Schilddrüsenkarzinom, hohes Rezidivrisiko bis zu 50 % innerhalb 10 Jahren bei gutartiger Struma multinodosa) versus einer totalen Thyreoidektomie (Funktionsverlust, Organverlust, Notwendigkeit der lebenslangen Substitutionstherapie, erhöhtes Risiko eines Hypoparathyreoidismus) sollte der Patient aufgeklärt werden. Dies ist im Operationsbericht bei der Indikationsbeschreibung dann explizit zu erwähnen [1–4]. Unter dem Aspekt einer hohen Rate an postoperativem Hypoparathyreoidismus propagieren wir, die prinzipielle Thyreoidektomie oder Hemithyreoidektomie, wie in den Leitlinien noch empfohlen, für manche Indikationen zu überdenken.

Auch bei der Behandlung des Schilddrüsenkarzinoms haben sich in den letzten Jahren völlig neue Aspekte ergeben. Durch ausgedehnte Qualitätsstudien des Outcomes nach Schilddrüsenoperation hat sich beispielsweise gezeigt, dass die totale Thyreoidektomie, insbesondere mit begleitender zentraler Kompartmentlymphadenektomie zu einer beträchtlichen Morbidität, speziell durch das Risiko des Hypoparathyreoidismus, führt [5, 6]. Für jeden arrivierten und routinierten Schilddrüsenchirurgen mit Verantwortungsbewusstsein ist dieses Problem nicht zu leugnen, wenngleich in der Literatur viele Autoren durch Selbstdarstellung optimierter Daten dieses Problem konsequent negieren. Der ehrliche Chirurg muss jedoch zugeben, dass das nicht der Realität entspricht, und Bergenfelz et al. hat mit seiner skandinavischen Registerstudie gezeigt, dass nach totaler Thyreoidektomie nahezu 5 % der Patienten an einer permanenten Nebenschilddrüsenunterfunktion leiden [7]. Aufgrund der Drop-Out Rate in der Langzeitbeobachtung dürfte die Dunkelziffer noch deutlich höher liegen. Dies sind wohl alarmierende Daten, die einerseits ein streng befundorientiertes Operationsverfahren einfordern und andererseits unangebrachte Radikalität verbieten. Die gekonnte Balance zwischen zurückhaltendem Resektionsausmaß und maximaler Radikalität zur Vermeidung von Rezidiven und folgenden Re-Eingriffen mit größerer kumulativer Komplikationsrate muss auf der Grundlage evidenzbasierter Daten erfolgen.

Ein entscheidender Fortschritt in der modernen Schilddrüsenchirurgie ist zweifelslos die zunehmende Minimierung eingriffstypischer Komplikationen. Die passagere **Recurrenspareserate** ist durch subtile mikrochirurgische und atraumatische Operationstechnik auf ein niedriges Maß reduziert worden. Einen wesentlichen Beitrag hat hier das intraoperative Neuromonitoring mit seinem ausgezeichneten negativen Vorhersagewert gebracht. Das bedeutet, dass bei regelrechtem Ableitungssignal bzw. einer adäquaten Amplitude von Nervus recurrens und Nervus vagus nach Entfernung eines Schilddrüsenlappens in über 95 % der Fälle von einer regulären Stimmbandfunktion auszugehen ist. Permanente Paresen stellen mit unter 0,5 % eine Seltenheit dar [8, 9]. Der **Hypoparathyreoidismus** ist nach wie vor ein Sorgenkind der Schilddrüsenchirurgen, speziell bei großen Strumen, bei der Autoimmunhyperthyreose und bei der zentralen Halsdissektion. Die Lokalisation der Nebenschilddrüsen kann äußerst exponiert sein. Sowohl das Erkennen als auch das vaskularisierte Erhalten der Epithelkörperchen kann selbst für den routinierten Schilddrüsenchirurgen eine Herausforderung darstellen [10, 11]. Die dritte eingriffstypische Komplikation, die **Nachblutung**, ist zu einem seltenen Ereignis geworden und wird deshalb im Zuge der modernen Aufklärung häufig vernachlässigt. Die Rate ist innerhalb der letzten Jahrzehnte durch subtile mikrochirurgische Operationstechniken und neue Operationsinstrumente zur Gewebeversiegelung deutlich zurückgegangen [12]. Im Einzelfall aber bleibt das akute Blutungsereignis durchaus dramatisch und erfordert rasches Handeln, nötigenfalls die sofortige Entlastung durch Wundöffnung am Krankenbett. „Standard operative procedures“, Schulung von Pflegepersonal und Ärzten *im fachübergreifenden Bereitschaftsdienst mit *regelmäßiger Bewusstmachung des Problems der Nachblutung sind daher unverzichtbar. *Jede Abteilung, die Schilddrüsenoperationen durchführt, sollte eine entsprechende SOP zum Management der Nachblutung bereitstellen.*

Zu bemerken ist, dass zur Optimierung der Ergebnisqualität einer endokrin-chirurgischen Abteilung stets eine lückenlose Erfassung der postoperativen aber auch permanenten Komplikationen in einer Datenbank notwendig ist. Qualitätsanalysen erlauben die Orientierung nach externen Daten und vorgegebenen Benchmarks. Die Auswertung der individuellen Einzelleistung der Chirurgen zeigt mitunter abweichende Komplikationsraten, selbst wenn diese als erfahren und spezialisiert gelten [12–14]. Bei der Optimierung des operateursspezifischen Outcome gilt es auch anzusetzen, um das Gesamtergebnis einer chirurgischen Abteilung zu optimieren und somit für eine Zertifizierung gewappnet zu sein.

## Die Bedeutung des Chirurgen bei der präoperativen Diagnostik, der Operationsindikation und der Operationsstrategie

Die erste Anlaufstelle für die Diagnostik und Therapie von Schilddrüsenerkrankungen ist traditionsgemäß der Nuklearmediziner oder Endokrinologe. Die initiale Erkennung eines Schilddrüsenproblems kann durch den Patienten selbst, den Hausarzt, Internisten, den HNO-Arzt oder durch den Radiologen, der den Schilddrüsenknoten als Zufallsbefund im Zuge bildgebender Verfahren für die Abklärung nicht thyreogener Krankheitsbilder erkennt, erfolgen. Funktionsstörungen können klinisch oder durch Abweichungen der TSH-Werte bzw. freien Schilddrüsenhormone im Zuge von Routinelaboruntersuchungen auffallen. Auch Gynäkologen und Geburtshelfer berücksichtigen immer mehr Funktionsstörungen der Schilddrüse oder auch Autoimmunerkrankungen.

**Figure 1:**
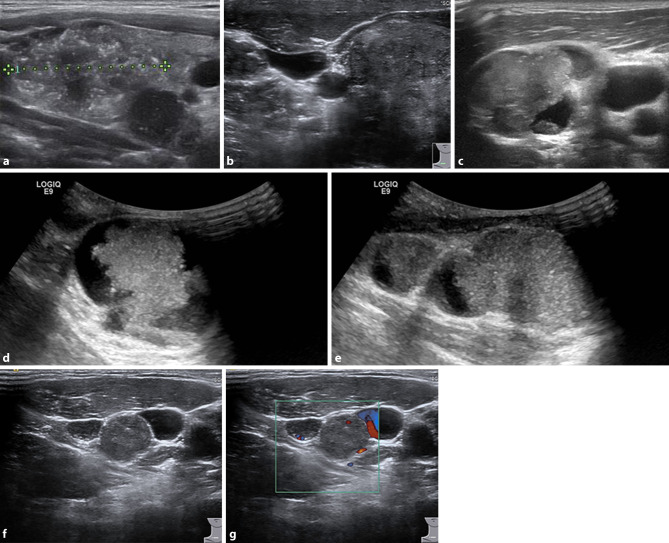

Ist nicht mit letzter Sicherheit ein beobachtendes Vorgehen angebracht oder ein konservatives Behandlungskonzept zielführend und steht eine Operationsindikation im Raum, so sollte bereits zu diesem Zeitpunkt der endokrin versierte Chirurg zugezogen werden. Es wäre jedem Schilddrüsenchirurgen zu empfehlen, auch selbst eine Sonographie der Schilddrüse und der Halslymphknoten durchzuführen. Papilläre Schilddrüsenkarzinome (Abb. 1a,b) und deren solide oder auch zystische Lymphknotenmetastasen (Abb. 1c–g) können typische Muster aufweisen und auf einen Blick zu erkennen sein. So kann der Chirurg beurteilen, welches Radikalitätsausmaß bzw. Operationsverfahren im individuellen Fall sinnvoll und notwendig ist. Auch die Labordiagnostik muss entsprechend berücksichtigt werden und in das operative Konzept miteinfließen, beispielsweise erhöhte Calcitoninwerte und Interpretation von Calciumstimulationstests oder Evaluierung von erhöhten Parathormonwerten zur Differenzierung eines primären oder eines sekundären Vitamin-D-mangelbedingten Hyperparathyreoidismus. Jedenfalls ist zur Planung der Operationsstrategie die Kenntnis der Schilddrüsenfunktion, der Schilddrüsenantikörper, des Calcitonins und der Nebenschilddrüsenparameter – Calcium, Parathormon und Vitamin D – von essentieller Bedeutung (Tab. 1). Damit verbunden ist auch die Verantwortung des modernen Schilddrüsenchirurgen, nicht nur mechanistisch das Handwerk der Thyreoidektomie möglichst gut und komplikationsfrei zu beherrschen, sondern ein umfassendes endokrin-chirurgisches Verständnis in seine operative Strategie einfließen zu lassen.

**Table 1:** 

Schilddrüsenlabor inklusive Antikörper, Thyreoglobulin optional
Calcitonin/Calciumstimulationstest falls Calcitonin erhöht, inkl. CEA
Serumcalcium, Parathormon, falls PTH erhöht Vit D
Sonographie inklusive lateraler Hals mit Beurteilung der Lymphknoten
Szintigraphie zur Erkennung von Autonomie und ektoper/retrosternaler Struma (mediastinales Fenster)
Feinnadelpunktion bei gezielter Indikation (Präselektion der Knoten durch Ultraschall und Szintigraphie)
CT-Hals Thorax ohne KM: Bei intrathorakaler Struma oder Verdacht auf mediastinaler/intrathorakaler Metastasierung
Bei Dyspnoe/großer Struma und Verdacht auf Organüberschreitung: MRT
HNO-Larynxbefund

**Figure 2:**
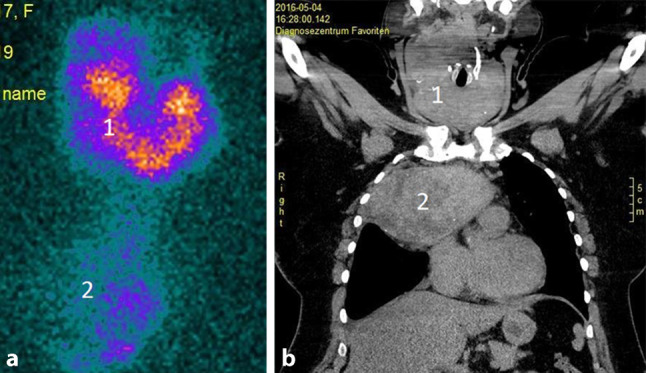

**Figure 3:**
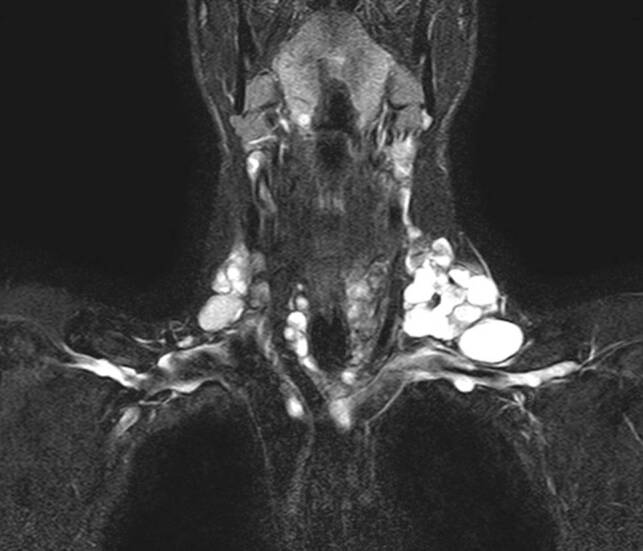

Auch obliegt es letztlich dem Chirurgen, die Ausdehnung einer Struma bereits präoperativ möglichst gut abzuklären. Bei Ausdehnung einer Schilddrüse in den Substernalraum bzw. fehlender sonographischer Abgrenzbarkeit nach caudal ist ergänzend ein Schnittbildverfahren zu wählen, das eine intrathorakale Ausbreitung oder auch isoliert intrathorakale Anteile erfasst und erkennen lässt, ob das vordere oder das hintere Mediastinum betroffen ist. Diese Information ist ganz wesentlich für die Operationsstrategie und die Frage, ob eine Erweiterung des Zugangsweges (z. B. Sternotomie) notwendig sein könnte. Die Szintigraphie kann hier erste Hinweise liefern, wenn das diagnostische Fenster in das obere Mediastinum erweitert wird (Abb. 2a). Als weiterführende Diagnostik ist ein CT ohne Kontrastmittel oft ausreichend (Abb. 2b). Jodhältige Kontrastmittel sind präoperativ grundsätzlich zu vermeiden, da im Falle von differenzierten Karzinomen verbliebene Schilddrüsen- oder Tumorzellen jodgesättigt sind und eine Radiojodtherapie dadurch nur verzögert möglich ist. Ein MRT kann speziell bei zystischen papillären Tumoren das Ausmaß einer Metastasierung in der T2-Gewichtung eindrucksvoll zeigen (Abb. 3) und ist darüber hinaus bei Verdacht einer Infiltration des Aerodigestivtraktes sehr zu empfehlen.

**Figure 4:**
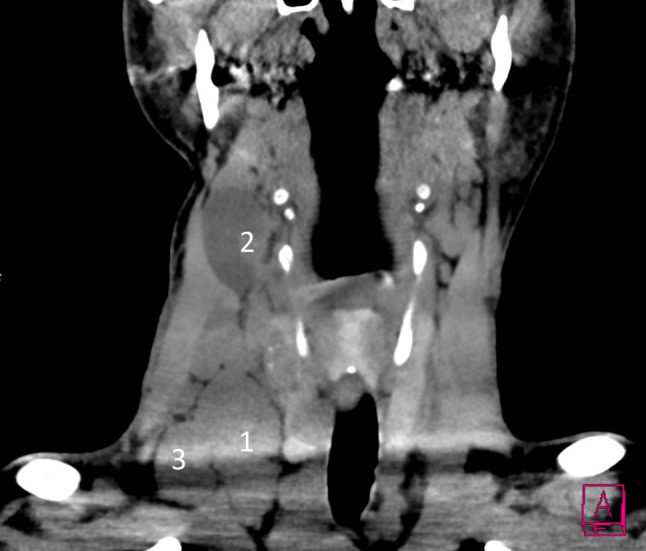

**Figure 5:**
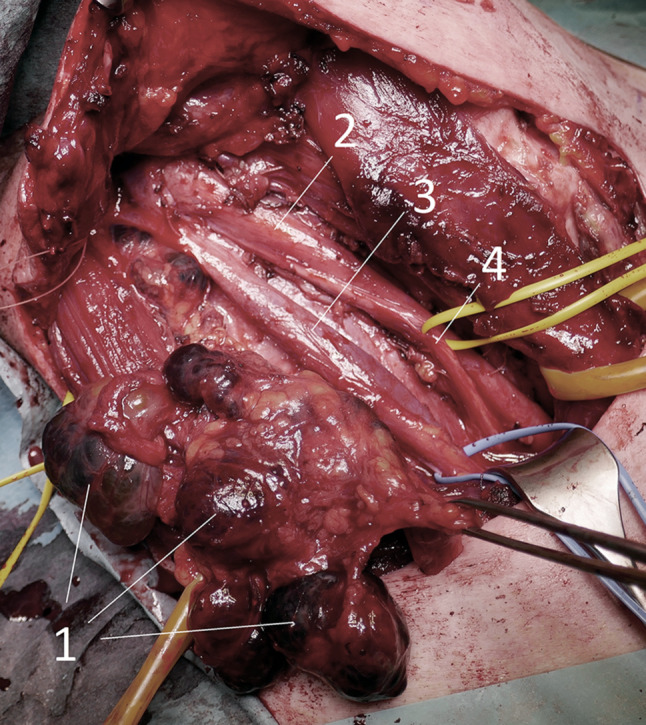

**Figure 6:**
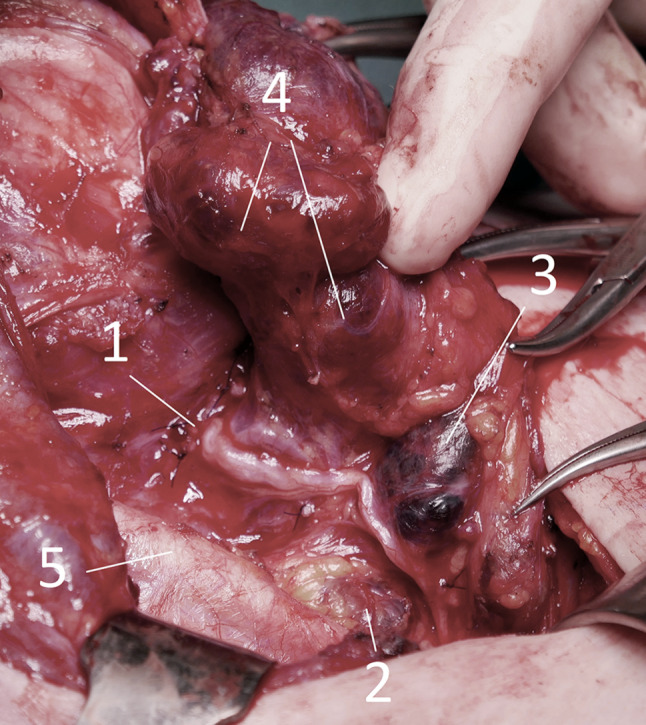

Die Ultraschalldiagnostik für Schilddrüse, Lymphknoten und Nebenschilddrüsen sollte demnach idealerweise auch vom Chirurgen beherrscht werden. Ein positiver Lymphknotennachweis im lateralen Kompartment (Regio IV, V, III, II – einen Überblick zeigt das CT in Abb. 4) lässt dann schon präoperativ eine laterale Halsdissektion einplanen (OP Bild Abb. 5), was sowohl für den Zugangsweg, die geplante Länge der Operation und auch die Wahl des spezialisierten Chirurgen von wesentlicher Bedeutung ist. Schwierig ist speziell die sonographische Lymphknotendiagnostik im zentralen Kompartment (Regio VI) bzw. im oberen mediastinalen Kompartment (Regio VII), die bei routinemäßiger Ultraschalluntersuchung durch Radiologen und Nuklearmediziner nicht immer erfasst wird. Anzumerken ist auch, dass retrotracheale, paraösophageale und vor allem sogenannte retrorecurrente (dorsal des N. laryngeus recurrens) Lymphknoten oft mit der Sonographie nicht einsehbar sind (Abb. 6; [15–17]).

Bei suspektem Lymphknotenbefund mit Mikrokalk, zystischen Veränderungen oder „schilddrüsenähnlichen“ Gewebsmustern kann in Kooperation mit dem zuweisenden Nuklearmediziner eine Feinnadelpunktion des Lymphknotens mit zytologischer Befundung oder Thyreoglobulin-wash-out durchgeführt werden.

Eine Feinnadelaspiration des Schilddrüsenknotens ist ebenfalls sehr aufschlussreich und kann dem Chirurgen signalisieren, ob er einen sparsamen oder großzügigen Zugangsweg wählen soll, ob er initial Lymphknoten mitentfernt oder ob möglicherweise eine eingeschränkte Knotenresektion möglich ist [18].

**Figure 7:**
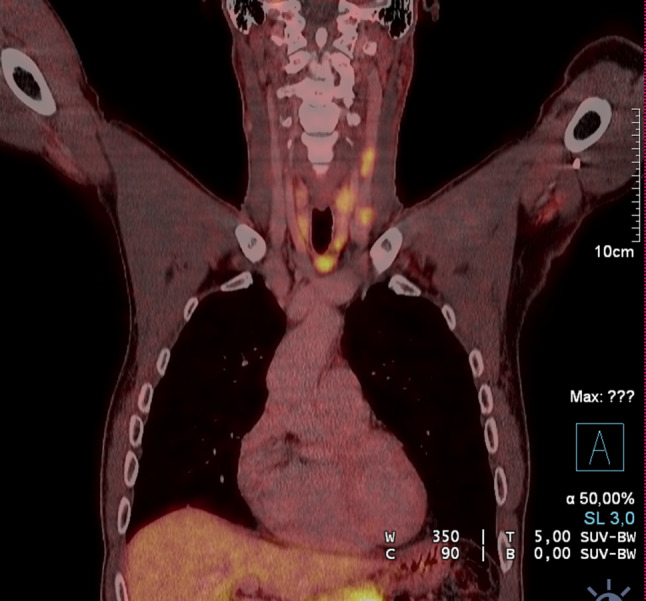

Inwieweit präoperativ zusätzliche nuklearmedizinische Untersuchungen oder eine nuklearmedizinisch-radiologische Fusionsdiagnostik zur Erfassung der Ausdehnung des Krankheitsbildes sinnvoll sind, muss interdisziplinär zwischen Chirurgen, Nuklearmedizinern und Radiologen besprochen werden. Bei medullären Schilddrüsenkarzinomen ist ein DOPA-PET-CT für die OP-Planung und das Resektionsausmaß jedenfalls hilfreich (Abb. 7). Insbesondere von Bedeutung ist die Ausnützung aller diagnostischen Möglichkeiten vor Re-Eingriffen.

Bei der Erstellung der Operationsindikation sind auch nicht thyreogene Faktoren zu berücksichtigen, beispielsweise eine kontralateral bestehende Recurrensparese, schwere Herz‑/Kreislauferkrankungen oder Koronarstents mit dualer Plättchentherapie, die üblicherweise im Zuge der ersten Bewertung der Schilddrüsenerkrankung nicht berücksichtigt werden.

Auch muss der Chirurg vor jeder Schilddrüsenoperation eine Funktionsabklärung der Nebenschilddrüsen einfordern. Vereinzelt liegt ein begleitender primärer Hyperparathyreoidismus vor und Calcium‑, Parathormon- und Vitamin D‑Werte sind selten Bestandteil der Routine-Schilddrüsendiagnostik. Nur so kann präoperativ eine Lokalisationsdiagnostik von möglicherweise adenomatösen Nebenschilddrüsen durchgeführt und im Zuge einer Thyreoidektomie auch gezielt das Epithelkörperchenadenom aufgesucht werden. Ein Apell, gleichzeitig die Nebenschilddrüsenfunktion abzuklären, sei hier auch an alle Schilddrüsendiagnostiker gerichtet.

## Die befundadaptierte Operation – die Wahl des Resektionsausmaßes

Der moderne Schilddrüsenchirurg muss die präoperative Diagnostik in sein Behandlungskonzept miteinbeziehen.

## Benigne Schilddrüsenerkrankungen

### Der solitäre Knoten – Operation auch funktionserhaltend möglich

**Figure 8:**
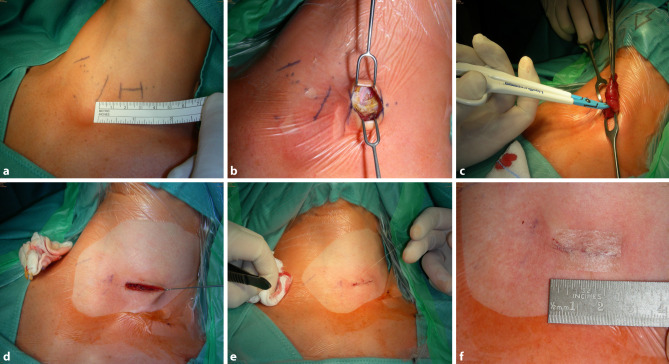

Besteht bei einem solitären Schilddrüsenknoten histologische Abklärungspflicht, so wird vielfach unkritisch als erster Schritt eine Hemithyreoidektomie gefordert, um eine primäre Sanierung des knotentragenden Schilddrüsenlappens zu erzielen [1, 19]. In vielen Fällen ist allerdings mit dem verbleibenden gesunden kontralateralen Schilddrüsenlappen keine sichere Euthyreose zu erzielen und der Patient wird substitutionspflichtig. Speziell bei jungen Patientinnen mit Kinderwunsch oder bei Schwangeren kann die notwendige Hormonreserve dann nicht ausreichen. Demnach ist eine gewebe-und funktionserhaltende Knotenresektion anzudenken, sofern der Schilddrüsenknoten günstig liegt (Abb. 8). Speziell trifft das für Knoten zu, die im oberen oder unteren Polbereich oder isthmusnahe lokalisiert sind. Bei diesem Resektionsverfahren ist eine Recurrenspräparation nicht notwendig bzw. die Nervenlokalisation mit Neuromonitoring möglich, ohne dass der Nervus laryngeus recurrens freigelegt werden muss. Somit kann zu jedem späteren Zeitpunkt ein komplikationsarmer Re-Eingriff bzw. die Komplettierungsoperation durchgeführt werden, sollte der Knoten im Schnellschnitt als gutartig beurteilt werden, sich allerdings in der definitiven Paraffinhistologie als Schilddrüsenkarzinom herausstellen. Liegt der zu entfernende Knoten unmittelbar in der Nähe des Nervus laryngeus recurrens, so ist eine komplette Freipräparation des Nerven notwendig, was zweifellos wiederum eine totale Lappenentfernung (Hemithyreoidektomie) erforderlich macht. In diesem Fall stellt sich allerdings die Frage, ob nicht gleich eine (zumindest ipsilaterale) zentrale Kompartmentlymphadenektomie (Halsdissektion) angeschlossen werden soll, da ein Re-Eingriff bei vorpräparierten Nervus laryngeus recurrens bzw. vorentfernten Schilddrüsenlappen tatsächlich die Morbidität erhöht. Hingegen ist eine Hemithyreoidektomie mit zentraler Lymphadenektomie bei vorangegangener eingeschränkter Knotenresektion unkompliziert. Somit ist eine primäre Hemithyreoidektomie bei gutartigem Solitärknoten nur notwendig, wenn der Knoten im dorsalen Lappenbereich liegt, einen Großteil des Lappens ausfüllt oder eine sehr suspekte Zytologie vorliegt [1, 3, 19].

### Struma multinodosa

Bei der Struma multinodosa ist tatsächlich eine Thyreoidektomie die Therapie der Wahl. Kritische Qualitätsanalysen und Ergebnisse aus Registerstudien zeigen allerdings eine bis zu 5 %-ige Rate an permanenten Hypoparathyreoidismus [2, 7, 14, 20, 21]. Diese Erkenntnis hat mittlerweile zu einem Umdenken geführt, sodass auch Dralle, der stets für radikalchirurgische Verfahren eingetreten ist, folgende Forderung stellt: „Surgery for endemic goiter—a plea for individualizing the extent of resection instead of heading for routine total thyroidectomy“ [22]. Demnach ist bei kritischer Position der Nebenschilddrüsen oder offensichtlicher Devaskularisierung auf der erstoperierten, befunddominanten Seite bei der operativen Vorgangsweise kontralateral besondere Vorsicht geboten und im Zweifelsfall ein kleiner Schilddrüsenrest als Schutzlamelle, die die Perfusion der Nebenschilddrüse sichert, zu belassen. Auch autotransplantierte Nebenschilddrüsen können eine suffiziente Nebenschilddrüsenfunktion nicht immer gewährleisten.

### Rezidivstruma

Der Hypoparathyreoidismus ist auch das Hauptproblem bei der Rezidivstruma, bei der zunächst streng nur die befunddominante Seite operiert werden soll, kleine unverdächtige und mechanisch nicht relevante Schilddrüsenknoten kontralateral sind zu belassen. Speziell bei Verdacht auf einen Schaden des Nervus laryngeus recurrens muss die Operation auf ein einseitiges Vorgehen limitiert werden. Die Verwendung des kontinuierlichen Neuromonitorings ist bei der Rezidivstruma empfohlen und hat sich gut bewährt. Die Operationszeit bei der Rezidivstruma kann durch die Anwendung des kontinuierlichen Neuromonitorings deutlich verkürzt werden [1, 19].

### Autoimmunhyperthyreose Morbus Basedow

Die (totale) Thyreoidektomie bei der Basedow Struma ist nach wie vor unstrittig. Die üblicherweise 18–24 Monate lange standardisierte konservative thyreostatische Therapie sollte einer früh elektiven Operation bei Vorliegen einer endokrinen Orbitopathie, großer Struma, ausgedehnter sonographisch verifizierter lymphomatöser Infiltrationen der Schilddrüse und extremer Vaskularisierung, gleichzeitig assoziierten Knoten, Schwangerschaftswunsch, Alter unter 40 Jahre und isolierte T3 Hyperthyreose (>40 % T3) weichen. Auch ist Patienten, bei denen sämtliche Schilddrüsenautoantikörper (neben TRAK auch TG-AK und aTPO) erhöht sind, eine zeitnahe definitive Sanierung anzuraten. Im Falle einer TRAK-Erhöhung >10 IU/L ist mit keiner dauerhaften Remission zu rechnen und die Thyreoidektomie frühzeititg ins Kalkül zu ziehen! Der GREAT („Graves’ Recurrent Event After Therapy“) Score einer holländischen Forschergruppe ermöglicht eine Risikostratifizierung (Tab. 2). In einer multizentrischen Studie wurde retrospektiv im Falle eines GREAT III (beispielsweise bei deutlich sichtbarer Struma, TRAK ≥6 IU/L , Serum fT4 (≥40 pmol/L), Alter <40 Jahre) das Rezidivrisiko von 73,6 % angegeben und daher der Vorteil einer primären Resektion hervorgehoben [23, 24]. Im Falle einer nicht therapierbaren Thyreotoxikose oder einer jodinduzierten Hyperthyreose kann eine dringliche Thyreoidektomie auch bei ausgeprägt manifester hyperthyreoter Stoffwechsellage unter Therapie mit Cortison und β‑Blocker mit geringem Risiko durchgeführt werden [19]. Hermann et al. haben nachgewiesen, dass es selbst bei florider Basedow-Hyperthyrose zu keiner intraoperativen Ausschüttung freier Schilddrüsenhormone kommt [25].

**Table 2:** 

	Clinical GREAT-Score	GREAT relatives Risiko für OP
*Alter (Jahre)*		0–1 = I 33,8 % 2–3 = II 59,4 % 4–6 = III 73,6 %
≥40	0	
<40	+1	
*Serum fT4 (pmol/l)*		
<40	0	
≥40	+1	
*Serum TRAK (iU/L)*		
<6	0	
6–19,9	+1	
≥20	+2	
*Strumagröße*		
0–I (nicht sichtbar bis tastbar in einer normalen oder gehobenen Kopfposition)	0	
II–III (sichtbar ab normaler Kopfposition)	+2	

### Thyreoiditis Hashimoto

Auch die Thyreoiditis Hashimoto kann ein chirurgisch zu therapierendes Krankheitsbild darstellen: Wenngleich zur Diskussion steht, ob die klassische Symptomatik von Hashimoto-Patienten tatsächlich thyreoidal bedingt ist, gibt es doch eindeutige Daten für eine Verbesserung der Lebensqualität nach Thyreoidektomie bei Patienten mit stark ausgeprägter Autoimmunerkrankung [26, 27]. Dies gilt für die hypertrophe Form der Hashimoto Thyreoiditis, bei der auch eine lokale mechanische Symptomatik bestehen kann. Eine weitere Indikation sind die oftmals beobachteten Hormonschwankungen, die eine konservative Führung der Patienten und eine stabile Euthyreose erschweren. So kann auch unerfüllter Kinderwunsch eine Indikation darstellen. Bei Vorliegen suspekter echoarmer Areale oder echoarmer Knoten ist die Chirurgie zur histologischen Abklärung indiziert, denn es kann äußerst schwierig sein, innerhalb des inhomogen, echoarmen Ultraschallmuster papilläre Karzinome auszuschließen oder zu erkennen [19].

### Thyreoiditis de Quervain

Sie ist eine seltene Erkrankung, typisch oftmals verbunden mit beträchtlichem lokalem Druckschmerz und ausgeprägtem Krankheitsgefühl. Die Erkrankung ist Domäne der konservativen Therapie mit antiphlogistischer Medikation und Cortisontherapie. Die Thyroiditis de Quervain ist möglicherweise eine Virusnacherkrankung, da man häufig fallende Virustiter findet. Sie gilt als selbstlimitiert und sollte in der Regel nach 6 Monaten ausgeheilt sein! Diesem Krankheitsbild liegt ein typisches flächig echoarmes wanderndes Ultraschallmuster zugrunde, das letztlich aber von infiltrativ wachsenden Malignomen nicht immer sicher zu diskriminieren ist. So ist die chirurgische Sanierung bei der Thyreoiditis de Quervain sowohl bei therapieresistenter Erkrankung als auch zur Sicherung der Diagnose und Ausschluss von Malignität durchaus ein Thema. Die Diagnostik ist bei typischer Klinik meist eindeutig, jedoch bei oligosymptomatischen Patienten oder Patienten mit einer silent Thyroiditis schwierig. Neben der chirurgischen Option zur Klärung dieser Fälle kann zunächst eine ultraschallgezielte Feinnadelpunktion hilfreich sein.

## Maligne Schilddrüsenerkrankungen

Beim Schilddrüsenkarzinom ist die chirurgische Therapie zunehmend differenzierter geworden. Hat man in früheren Jahren grundsätzlich thyreoidektomiert und beidseits zentral lymphadenektomiert (=zentrale Halsdissektion), die lateralen jugulären Lymphknoten im Level IV im Schnellschnitt untersucht und bei Befall das laterale Kompartiment komplett disseziert, so geht man heute risikoadaptierte Wege [3, 5, 28]. Das betrifft sowohl das Risiko eines potenziellen Rezidivs, als auch das chirurgische Komplikationsrisiko. So haben klinische Langzeitbeobachtungen auch dazu geführt, dass die Pathologen ihre Klassifikation korrigieren: Als jüngstes Beispiel gilt das **NIFTP** (**Noninvasive follicular thyroid neoplasm with papillary-like nuclear features)**, ursprünglich als abgekapselte follikuläre Variante des papillären Karzinoms klassifiziert, nun mehr ein nicht als Krebserkrankung einzustufender Tumor, der unabhängig von seiner Größe keine obligate Thyreoidektomie, keine Lymphadenektomie und keine Radiojodtherapie erforderlich macht, da er praktisch nie metastasiert.

Das **papilläre Schilddrüsenkarzinom** von über 1 cm Größe (größer pT1a) erfordert hingegen eine Thyreoidektomie mit zumindest ipsilateraler Kompartmentlymphadenektomie, bei negativer Schnellschnittdiagnose der Lymphknoten kann eine kontralaterale zentrale Halsdissektion unterbleiben, speziell wenn dies ein Risiko für eine intakte Nebenschilddrüsenfunktion darstellt [3, 5, 29]. Eine Lymphknotenbiopsie im lateralen Kompartment (Regio IV) wird zunehmend verlassen, wenn sonographisch kein positiver Lymphknotenbefund vorliegt. Sonographisch nicht erkennbare Mikrometastasen beeinträchtigen die Prognose des papillären Schilddrüsenkarzinoms nicht [30]. Sollte im Langzeitverlauf ein Lymphknotenrezidiv im lateralen Kompartment auftreten, so ist eine laterale Halsdissektion im nicht-voroperierten Bereich auch im Intervall komplikationslos möglich, da sich die wesentlichen anatomischen Strukturen, wie die Vena jugularis, der Nervus vagus, der Nervus phrenicus etc. in unberührter chirurgischer Schicht gut freilegen lassen [29].

Auch bei Multifokalität von Mikrokarzinomen sollte nicht reflexartig ein radikaltherapeutisches Konzept eingeschlagen werden, vor allem, da dieser Befund meist erst postoperativ im Paraffinschnitt detektiert wird und ein Re-Eingriff abzuwägen ist [31]. Schließlich bestehen mittlerweile einschlägige Trends, Mikrokarzinome überhaupt nur zu observieren [32–34]. In solchen Situationen muss die Entscheidungsfindung interdisziplinär getroffen werden und der Beitrag des Chirurgen zur Risikoabwägung ist dabei von wesentlicher Bedeutung.

Auch die **follikulären Karzinome** wurden vom Pathologen zunehmend differenziert eingestuft: Das minimal invasive follikuläre Schilddrüsenkarzinom (MIFTC) mit ausschließlicher Kapselinvasion des Tumors bedarf lediglich einer Hemithyreoidektomie. Erst bei Angioinvasion ist eine totale Thyreoidektomie empfohlen [35]. Die Indikation zur Radiojodtherapie kann vom postoperativen TG-Verlauf abhängig gemacht werden [3]. Eine Lymphknotenchirurgie ist definitiv nicht indiziert, eine zentrale Kompartmentlymphadenektomie abzulehnen und bei eintretender Komplikation kaum zu begründen. Zervicale Lymphknotenmetastasen treten nur bei weit invasiven follikulären Karzinomen im fortgeschrittenen bereits fernmetastasierten Stadium auf, welche dann klinisch nicht mehr relevant sind [5].

Das **medulläre Schilddrüsenkarzinom** erfordert grundsätzlich eine Thyreoidektomie mit zentraler Kompartmentlympadenektomie (Level VI und VII). Doch auch hier fließen Kriterien wie beispielsweise die desmoplastische Stromareaktion in die weitere Strategie mit ein: Desmoplasie-negative Tumore zeigen keine Lymphknotenmetastasierung, weshalb auf eine Neck-Dissection (laterale Halsdissektion) verzichtet werden kann [36, 37]. Diese Desmoplasie ist von erfahrenen Pathologen bereits im Schnellschnitt erkennbar. Durch die kurze Halbwertszeit des Calcitonins ist bereits nach wenigen Tagen zu erkennen, ob biochemisch nachweisbare Tumorfreiheit erreicht wurde oder eventuell eine Erweiterung der Operation in den lateralen Hals notwendig wird. Dabei kann als Entscheidungshilfe auch das DOPA-PET dienen um zu erkennen, ob nicht eine Fernmetastasierung (bevorzugt in der Leber) Ursache für ein persistierendes Calcitonin ist. Lebermetastasen können allerdings auch diffus auftreten und eine Lebermetastasierung somit nicht erkennbar sein. Dies würde nur durch eine blinde Leberbiopsie zu diagnostizieren sein [5].

## Komplikationsvermeidung

Galt früher die Recurrensparese als DIE eingriffstypische Komplikation bei Schilddrüsenoperationen und die Nachblutung als einzig vital bedrohende Folge, so steht heute der Hypoparathyreoidismus bzw. seine Vermeidungsstrategien im Fokus der modernen Chirurgie.

### Recurrensparese

Durch subtile mikrochirurgische Operationstechniken einerseits, durch Anwendung des intraoperativen Neuromonitoring (IONM) andererseits ist die Rate der Recurrensparesen während der letzten Jahrzehnte deutlich zurückgegangen. Aktuell kann man von einer postoperativen Pareserate zwischen 2 und 5 %, je nach Schwierigkeitsgrad des Eingriffs, ausgehen. Ein zusätzliches Qualitätskriterium ist, dass die Rückbildungsrate bei eingetretener Stimmbandnervlähmung bei ordnungsgemäß durchgeführter Operation über 90 % beträgt und demnach die permanente Recurrensparese (definitionsgemäß bestehend über 6 Monate) mit 0,5 % zur Rarität geworden ist [4, 8, 38–40]. Eine wesentliche Strategie zur Vermeidung der dramatisch verlaufenden beidseitigen Recurrensparese ist das zweizeitige Vorgehen [41–43], das mittlerweile auf breite Akzeptanz bei den Chirurgen und Verständnis bei den darüber präoperativ aufgeklärten Patienten gestoßen ist: Bei Ausfall der Nervenleitung am erstoperierten Schilddrüsenlappen wird der Eingriff nach Entfernung der ersten Seite beendet und die Komplettierung erst durchgeführt, wenn sich die Stimmbandbeweglichkeit wieder erholt hat. Ausgenommen von dieser Vorgangsweise sind jene Patienten, bei denen aus onkologischen Gründen eine Nervenresektion an der tumortragenden Seite erfolgen muss, da eine Chance auf Rückbildung der Parese nicht mehr gegeben ist. Da der negative Vorhersagewert (negative predictive value – NPV) sehr hoch ist (96 bis 98 %), kann man sich bei regulärem Abschlusssignal nach Entfernung des befunddominanten Schilddrüsenlappens auf eine postoperativ normale Stimmbandfunktion verlassen und dadurch abgesichert auch die kontralaterale Seite operieren. Bei unsicherem Neuromonitoringsignal oder Ausfall im Sinne eines LOS (Loss of Signal) Typ I (lokaler Nervenausfall) oder II (globaler Nervenausfall) wird die Stimmbandfunktion am ersten postoperativen Tag geprüft. Bei intakter Beweglichkeit kann mit nur ein- bis zweitägiger Verzögerung die Komplettierung erfolgen. Bei Vorliegen einer Recurrensparese wird die Rekonvaleszenz der Nervenfunktion abgewartet. Mit dieser Strategie ist die klinisch dramatische Komplikation einer beidseitigen Recurrensparese nahezu auszuschließen.

Grundsätzlich muss die Definition der „Recurrensparese“ verfeinert werden: Unsere Arbeitsgruppe konnte anhand einer großen Fallzahl zeigen, dass man zwischen einer „kompletten“ und „inkompletten“ Parese, demnach zwischen Stimmbandstillstand und Minderbeweglichkeit unterscheiden muss; letztere verläuft klinisch milder und garantiert eine rasche Rückbildung [44].

Zu wenig Beachtung wird dem Ramus externus des Nervus laryngeus superior geschenkt. Seine Läsion führt zu einem Verlust der Obertöne und des Stimmvolumens, der Nerv unterliegt anatomischen Varianten [45]. Er kann zwischen den Ästen der A. thyreoidea superior hindurchlaufen und bei der Versorgung der oberen Polgefäße geschädigt werden. Das IONM ist bei der Identifikation dieses Nervenastes, seines Verlaufs und seiner Funktion sehr hilfreich.

### Hypoparathyreoidismus

Der postoperative Hypoparathyreoidismus ist nach wie vor das größte Problem bei totaler Thyreoidektomie oder Rezidiveingriffen. Speziell bei zentraler Kompartmentlymphadenektomie kann die Nebenschilddrüsenfunktion gefährdet sein, vor allem wenn die Nebenschilddrüsen in äußerst exponierter schilddrüsennaher Position liegen oder auch untrennbar mit der Schilddrüsenkapsel verbunden sind, sodass es in einigen Fällen auch nicht möglich ist, Epithelkörperchen gut vaskularisiert zu erhalten. In sieben Prozent der Fälle wird die Nebenschilddrüse komplett von den Gefäßen der Schilddrüse versorgt, weshalb solche Nebenschilddrüsen in jedem Fall autotransplantiert werden müssen. Alternativ ist abzuwägen, ob der epithelkörperchentragende Schilddrüsenanteil belassen werden kann [46]. Eine Anzahl dargestellter Nebenschilddrüsen, die zur sicheren Vermeidung des Hypoparathyreoidismus erforderlich ist, ist nicht definierbar, da die Position der Epithelkörperchen einer großen Variationsbreite unterliegt – oft auch in deutlicher Distanz zur Schilddrüse bzw. auch außerhalb der Schilddrüsenloge und somit nicht identifizierbar [10]. Dieser Tatsache sind sich Chirurgen immer wieder bewusst, wenn aufgrund eines primären Hyperparathyreoidismus ein zu entfernendes Nebenschilddrüsenadenom nicht an typischer Stelle liegt. Der Leitspruch des Autors ist: „Man muss nicht jede Nebenschilddrüse sehen, man darf jedoch keine übersehen“. Nach Möglichkeit sollten exponiert liegende Nebenschilddrüsen unter Schonung ihrer Vaskularisierung erhalten bleiben, wobei auch eine zarte vaskularisierte Bindegewebsbrücke ausreicht, langfristig die Funktion der Nebenschilddrüse zu erhalten. Auch eine intraoperative Verfärbung ist kein Anlass für eine Exstirpation mit konsekutiver Autotransplantation [47].

**Figure 9:**
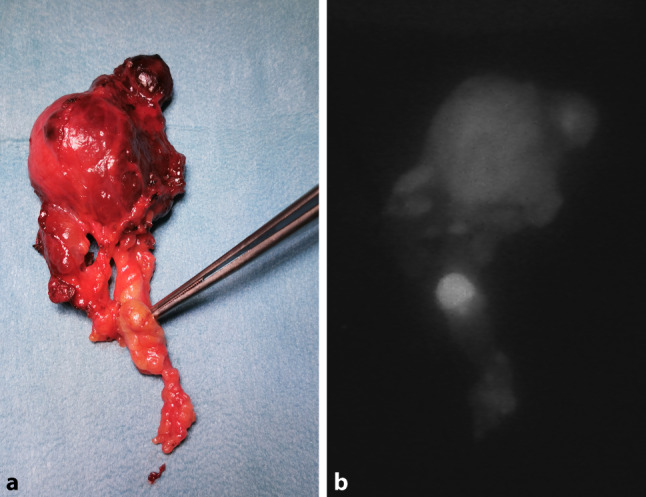

Bei Nichtauffinden einer Nebenschilddrüse im OP-Situs ist allerdings das resezierte Schilddrüsenpräparat im Kapselbereich mikrochirurgisch zu dissezieren, um unbeabsichtigt entfernte Nebenschilddrüsen zu entdecken und einer Autotransplantation zuzuführen. Vor allem ist die Begutachtung des Präparats am OP Tisch bei en bloc Thyreoidektomie mit zentralem Lymphknotenkompartement wichtig, da darin Nebenschilddrüsen verborgen sein können. Bei der Problematik der Nebenschilddrüsendarstellung kommt in Zukunft die vielversprechende Methode der Autoimmunfluoreszenzdarstellung auf uns zu, wobei man sich die Eigenschaft der Nebenschilddrüsen zunutze macht, bei Beleuchtung mit definiertem Laserlicht zu fluoreszieren und so mit einer speziellen Kamera sichtbar zu werden (Abb. 9a,b; [48, 49]). Zusätzlich kann man durch intraoperative Injektion von ICG (Indocyaningrün) auch die Durchblutung der Epithelkörperchen prüfen [50–52]. Die korrekte Technik der Autotransplantation setzt ein Morcellement der Nebenschilddrüse in möglichst kleine Fragmente voraus, da das Nebenschilddrüsengewebe anfänglich nur durch Diffusion ernährt wird und diese erreicht nur 6 Zelllagen. 80 % der korrekt autotransplantierten Nebenschilddrüsen produzieren langfristig wieder Parathormon [53].

### Nachblutung

Aufgrund der zunehmend subtilen mikrochirurgischen Operationstechnik und der selektiven Gefäßversorgungen ist die Nachblutungsrate beträchtlich zurückgegangen und liegt bei unter 1 %. Dennoch besteht im Eintrittsfall hohe Alarmbereitschaft, da eine zu späte Erkennung und ein verzögertes Eingreifen im Sinne einer Revisionsoperation zu einer lebensbedrohlichen Situation führen kann. Höchste Dringlichkeit zur Wundöffnung besteht bei Atemnot. Im dramatisch verlaufenden Fall muss auch eine Wundöffnung direkt am Bett erfolgen und dabei nicht nur die Hautnaht, sondern auch Adaptationsnähte der Subcutis und der infrahyoidalen Muskulatur durchtrennt werden, andernfalls wird die tiefe Schilddrüsenloge nicht entlastet. Trotz des seltenen Vorkommens bleibt sie die einzige potenziell lebensbedrohliche Komplikation in der Schilddrüsenchirurgie. Es hat sich aber gezeigt, dass bei unauffälligem postoperativem Verlauf das Nachblutungsrisiko nach 24 h kaum mehr vorhanden ist [54]. Somit ist eine eintägige postoperative stationäre Überwachung notwendig und eine ambulante Schilddrüsenchirurgie abzulehnen.

## Neue extrazervikale Zugänge zur Vermeidung der Narbe am Hals – minimal invasiv oder maximal invasiv?

In den letzten Jahren haben mehrere extrazervikale videoendoskopische Zugänge zur Schilddrüse von sich hören gemacht, die ausschließlich dazu dienen, die Narbe am Hals zu vermeiden. Transaxilläre-transmammäre (ABBA), retroauriculäre (Endocats) oder transorale Zugänge haben sich entwickelt, die aufgrund des distanten Zugangsweges größere Wundflächen nach sich ziehen als die direkte Kocher’sche Inzision im Bereich der Schilddrüse [55–57]. Im Gegensatz dazu hat die laparoskopische minimal invasive Chirurgie im Abdomen entscheidende Vorteile gebracht, war doch zuvor das Verhältnis der Hautincision im Vergleich zum Operationsfeld im Bauchraum unverhältnismäßig groß (z. B. Nebenniere oder Gallenblase). Beim extrazervikalen Zugang zur Schilddrüse wird genau der umgekehrte Weg beschritten (größere Wundfläche, mehr Gewebemobilisation). Die Entfernung der Schilddrüse wird ausschließlich mit Hitzekoagulation durchgeführt und es müssen erst größere Studien beweisen, dass die Ergebnisqualität dieser Eingriffe mit der etablierten Operationstechnik mithalten kann. Das betrifft sowohl die Radikalität des Eingriffs, als auch die niedrigen Komplikationsraten. Durch diese Zugangswege sind auch bisher eingriffs-untypische Komplikationen eingetreten, die sowohl auf den extraanatomischen Zugangsweg, als auch auf die dafür notwendige Lagerung des Patienten zurückzuführen sind [58]. Speziell die transorale Operation, die neuerdings im Vormarsch ist, muss in Zentren erfolgen, unter Studienbedingungen stattfinden und einer strengen Qualitätskontrolle unterliegen. Etablierte minimal-invasive Verfahren stellen die MIVAT (minimal invasive videoassisierte Thyreoidektomie) bzw. die OMIT (offene minimal invasive Thyreoidektomie) dar, die mit einer kleinstmöglichen Inzision an typischer Stelle auskommen, welche bei Bedarf etwas erweitert werden kann („Tailored Incision“). Grundsätzlich zu erwähnen ist, dass selbst bei jungen Patienten mit faltenfreiem Hals eine symmetrisch positionierte Narbe langfristig in den meisten Fällen kaum mehr erkennbar bleibt. Eine Voraussetzung dafür ist, dass der Chirurg präoperativ den Hautschnitt am sitzenden Patienten markiert und die optimale Inzisionshöhe – idealer Weise in einer präformierten Hautfalte – für den jeweiligen Eingriff erkennt und markiert. Es muss auch untersucht werden, ob der kosmetische Vorteil durch die extrazervikalen oder transoralen Operationsmethoden im Vergleich zu den Standardmethoden langfristig signifikant gegeben ist.

Zur Alternative der Radiofrequenzablation: für gewisse OP-Indikationen wie Zysten und autonome Adenome, gutartigen Knoten mit lokalen Beschwerdebild oder bei Kontraindikation zur Operation kann die Radiofrequenzablation in Erwägung gezogen werden. Zu bedenken ist allerdings, dass die Knoten nicht zur Gänze entfernt, sondern nur in ihrer Größe reduziert werden können [59].
